# Targeting autophagy overcomes Enzalutamide resistance in castration-resistant prostate cancer cells and improves therapeutic response in a xenograft model

**DOI:** 10.1038/onc.2014.25

**Published:** 2014-03-24

**Authors:** H G Nguyen, J C Yang, H-J Kung, X-B Shi, D Tilki, P N Lara, R W DeVere White, A C Gao, C P Evans

**Affiliations:** 1 Department of Urology, UC Davis School of Medicine, Sacramento, CA, USA; 2 Department of Biochemistry and Molecular Medicine, UC Davis School of Medicine, Sacramento, CA, USA; 3 UC Davis Comprehensive Cancer Center, UC Davis School of Medicine, Sacramento, CA, USA

## Abstract

Macro-autophagy is associated with drug resistance in various cancers and can function as an adaptive response to maintain cell survival under metabolic stresses, including androgen deprivation. Androgen deprivation or treatment with androgen receptor (AR) signaling inhibitor (ARSI), Enzalutamide (MDV-3100, ENZA) or bicalutamide induced autophagy in androgen-dependent and in castration-resistant CaP (castration-resistant prostate cancer (CRPC)) cell lines. The autophagic cascade triggered by AR blockage, correlated with the increased light chain 3-II/I ratio and ATG-5 expression. Autophagy was observed in a subpopulation of C4-2B cells that developed insensitivity to ENZA after sustained exposure in culture. Using flow cytometry and clonogenic assays, we showed that inhibiting autophagy with clomipramine (CMI), chloroquine or metformin increased apoptosis and significantly impaired cell viability. This autophagic process was mediated by AMP-dependent protein kinase (AMPK) activation and the suppression of mammalian target of rapamycin (mTOR) through Raptor phosphorylation (Serine 792). Furthermore, small interfering RNA targeting AMPK significantly inhibited autophagy and promoted cell death in CaP cells acutely or chronically exposed to ENZA or androgen deprivation, suggesting that autophagy is an important survival mechanism in CRPC. Lastly, *in vivo* studies with mice orthotopically implanted with ENZA-resistant cells demonstrated that the combination of ENZA and autophagy modulators, CMI or metformin significantly reduced tumor growth when compared with control groups (*P*<0.005). In conclusion, autophagy is as an important mechanism of resistance to ARSI in CRPC. Antiandrogen-induced autophagy is mediated through the activation of AMPK pathway and the suppression of mTOR pathway. Blocking autophagy pharmacologically or genetically significantly impairs prostate cancer cell survival *in vitro* and *in vivo*, implying the therapeutics potential of autophagy inhibitors in the antiandrogen-resistance setting.

## Introduction

Progression to castration resistance is almost universal in patients with metastatic prostate cancer after a period androgen-deprivation therapy. Castration-resistant prostate cancer (CRPC) continues to account for >28 000 deaths each year in the United States. Studies have demonstrated that persistent androgen receptor (AR) signaling remained the key driver in the progression to CRPC. CRPC cells acquired the ability to activate AR signaling either through AR gene amplification, AR mutation, constitutively active AR splice variants or increased intratumor androgen production in the setting of androgen-deprivation therapy. New generation of AR signaling inhibitors (ARSIs), Enzalutamide (MDV-3100, ENZA), a small molecule with multiple effects on androgen signaling, including blockage of testosterone binding to AR, preventing AR nuclear translocation and DNA binding, and interfering with co-activator recruitment, gave patients who failed docetaxel a 4.8-month survival benefit over placebo in the AFFIRM Phase III trial.^1^ It is also known that not all patients responded to the new generation of ARSIs and continued to progress and die from the disease. Finding the mechanism of resistance to the new ARSI therapy and aiming at delaying the progression of CRPC has been the objectives of many laboratories, including ours.

Numerous mechanisms of resistance or survival in which prostate cancer cells use to counter the effects of androgen ablation or antiandrogen therapy have been described.^2^ Autophagy or self-eating is a catabolic process that is activated in response to stresses, allowing cells to use the lysosomal-mediated degradation of cellular proteins and organelles to regenerate energy. Autophagy is constitutively active in cells at a basal level to help maintain cellular homeostasis by eliminating aged cellular components. Cancer cells use autophagy to prolong their survival under harsh conditions of metabolic stress in the tumor microenvironment induced by chemotherapy, ionizing radiation, nutritional starvation, oxidative stress or growth factor deprivation.^3^ On the other hand, excessive or deregulated autophagy can push the cells toward autophagic cell death or type-II programmed cell death and is thought to have a role in tumor suppression. Modulating autophagy has recently been exploited as a molecular target to improve cancer therapy.

Regulation of autophagy involves multiple pathways: (1) under nutrient-rich conditions, the phosphatidylinositol 3′ kinase (PI3K; type I)/Akt pathway inhibits autophagy through the activation of mammalian target of rapamycin (mTOR); (2) under nutrient or growth factor-deprived or hypoxic conditions, autophagy is activated by the AMPK (AMP-dependent protein kinase) pathway, leading to the upregulation of transcription of autophagy genes as well as the suppression of mTOR/S6K/4EBP activity by phosphorylation of TSC2.^4^ The activation of AMPK as well as the suppression of PI3K/AKT/ mTOR are the main triggers of autophagy induction, leading to the multistep process of autophagosome formation around cellular organelles targeted for degradation. The autophagosome then fuses with lysosomes where contents are degraded into amino acid, fatty acid and nucleotides to be reused. The autophagosome is formed by Atg5 and Atg12 conjugation, which then recruits microtubule-associated protein light chain 3-II (LC3-II) for insertion into autophagosome membranes. The LC3- eGFP-transgene has been exploited to provide a reliable visual marker to detect autophagic process.^4^

Accumulating evidence showed that targeting autophagy alone or in combination with chemotherapy has been effective at enhancing cell death and improved the efficacy of the cancer therapies in a variety of cancer types.^3^ Our group has previously shown that inhibiting autophagy enhances cell-killing effects of Src inhibitors in CaP.^5^ We hypothesized that autophagy is a survival mechanism that prostate cancer cells used to evade the insult of androgen deprivation or antiandrogen therapy, including the new generation of ARSI, ENZA. Inhibiting autophagy as a molecular target could overcome resistance to ENZA therapy in CRPC.

Previous studies by Jiang *et al.*^6^ demonstrated that knocking down AR in LNCaP or CWR22Rv1 led to increased autophagy. Androgen deprivation in the setting of hypoxia also induced autophagy.^7,8^ Most recently, Boutin *et al.*^9^ demonstrated that androgen deprivation or treatment with bicalutamide in LNCaP cells induced autophagy and inhibiting autophagy by small interfering RNA (siRNA) to ATG-5 sensitized LNCaP cells to bicalutamide-induced apoptosis. However, the mechanism of antiandrogen-induced autophagy is still poorly understood. The question of whether autophagy has an important role in mediating resistance to the new ARSI ENZA has not been elucidated *in vitro* or *in vivo*.

In the present study, we showed that blocking AR axis by androgen deprivation or treatment with ARSI ENZA or bicalutamide induced autophagy in androgen-dependent and in CRPC cell lines. Autophagy was observed in a subpopulation of C4-2B cells that developed insensitivity to ENZA after sustained exposure in culture. This autophagic process was mediated by AMPK activation and the suppression of mTOR through Raptor phosphorylation (Serine 792). Furthermore, siRNA targeting AMPK significantly inhibited autophagy and promoted cell death in CaP cells acutely or chronically exposed to ENZA or androgen-deprived culturing condition, suggesting that autophagy is an important survival mechanism in CRPC. Lastly, *in vivo* studies with mice orthotopically implanted with ENZA-resistant cells demonstrated that the combination of ENZA and autophagy modulators clomipramine (CMI) or metformin significantly reduced tumor growth when compared with control groups.

## Results

### AR signaling inhibition by ENZA induces autophagy in both androgen-responsive and CRPC cells

**Figure 1:**
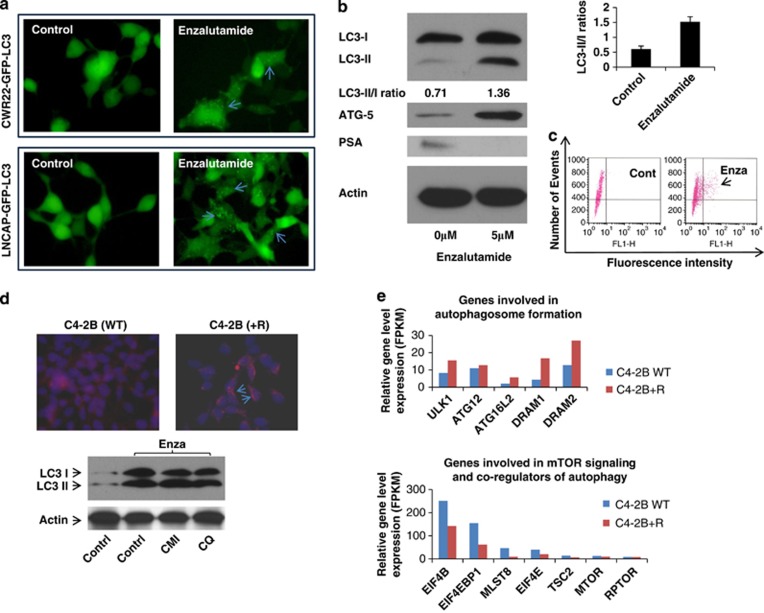
(**a**) AR inhibition by ENZA induces autophagy in both androgen-responsive and CRPC cell lines. Representative fluorescence microscopy of CWR22Rv1-eGFP-LC3 (upper panel) and LNCaP-eGFP-LC3 (lower panel) cells showing GFP-LC3 localization and puncta autophagosome formation represented by arrow. Stable cell lines expressing eGFP-LC3 were treated with DMSO (vehicle control), 10 μM ENZA for 48 h and were then analyzed by fluorescence microscopy. (**b**) Inhibition of AR by ENZA-induced autophagy. C4-2B and LNCaP cells were treated with 5 μM ENZA, and cell lysates were harvested and subjected to western blotting analysis using autophagy markers, LC3-I and LC3-II and ATG-5. PSA was used as internal control. β-Actin was used as the loading control. (**c**) Demonstration of quantification of autophagosomes using flow cytometry. Upon induction of autophagy, eGFP-LC3-transfected cells were gated and numbered to report the degree of autophagy. (**d**) Chronic exposure to ENZA resulted in autophagy. To mimic resistant to ENZA, C4-2B cells were subjected to 20 μM ENZA over a period of 3 months and selected for a sub-population of resistance cells (C4-2B+R). Upper panel showed acridine orange staining of autophagosome acidic vesicles as a marker for autophagy. Lower panel showed LC3-I/II protein expression in parental cells (control) and under chronic ENZA exposure-treated vehicle control, chloroquine (CQ) or CMI. β-Actin was used as the loading control. (**e**) Transcriptome deep sequencing of parental and ENZA-resistant CRPC cell lines. Total RNA was extracted from C4-2B parental (C4-2B-WT) and ENZA-resistant (C4-2B+R) cells subjected to next-generation deep RNA sequencing and bioinformatics analysis. The levels of autophagy gene transcripts were expressed in FPKM (3 FPKM=1 transcript/cell). Examples of genes involved in autophagosome formationand mTOR signaling are shown.

To determine whether both androgen responsive (LNCaP) and androgen insensitive (CWR22Rv1) cell lines undergo autophagy during AR signaling inhibition by ENZA, both cell lines stably introduced with LC3-eGFP were treated with 10 μM of ENZA. LC3-I in cells is localized in the cytosol, but upon induction of autophagy, it is lipidated into LC3-II and inserted into autophagosome membrane and can readily be detected and visualized by the prominent change from diffuse cytoplasmic to bright, punctate fluorescence in the cytosol as shown in Figure 1a. Additional evidence of ENZA-induced autophagy in LNCaP and CWR22Rv1 cells came from western blotting analysis as demonstrated by the significant increase in the LC3-I to LC3-II conversion (LC3-II/I ratio increased from 0.71 to 1.36) and the increased expression of ATG 5, both have been used as reliable markers of autophagy (Figure 1b).^4^ Flow cytometry was used to measure and quantify increase of autophagosome formation upon ENZA treatment as shown in Figure 1c. To model resistance to ENZA *in vivo*, C4-2B cells were treated with ENZA at 20 μM for a period of 3 months and selected for cells that survived after this prolonged drug exposure. ENZA-resistant C4-2B cells were examined for the presence of autophagy using acridine orange staining and western blotting analysis. As showed in Figure 1d, cells resistant to chronic ARSI by ENZA displayed an increased level of basal autophagy. C4-2B+R cells also harbored a higher LC3-II/I ratio than the parental cells. These C4-2B+R cells retain the same T877A mutation in AR as in their parental LNCaP line but not F876L mutation as in the other spontaneous ENZA-resistant LNCaP cells (data not shown).^10^ Transcriptome deep sequencing of parental and ENZA-resistant C4-2B cells was carried out to examine differential gene expression pattern that may be related to their ability to survive under constant high exposure to ARSI. More than 140 genes were upregulated and >100 genes were downregulated, (3 FPKM (fragments per kilobase of exon per million mapped fragments)=1 transcript/cell). Figure 1e showed examples of upregulated genes involved in autophagosome formation as expected in the ENZA-resistant cells. Suppression of mTOR signaling is also a major inducer of autophagy. We observed several mTOR signaling genes that were differentially downregulated in the resistant cells when compared with the parental cells (expression levels are expressed in FPKM with 3 FPKM=1 transcript/cell).

### AR signaling inhibition-mediated autophagy is a class effect

**Figure 2:**
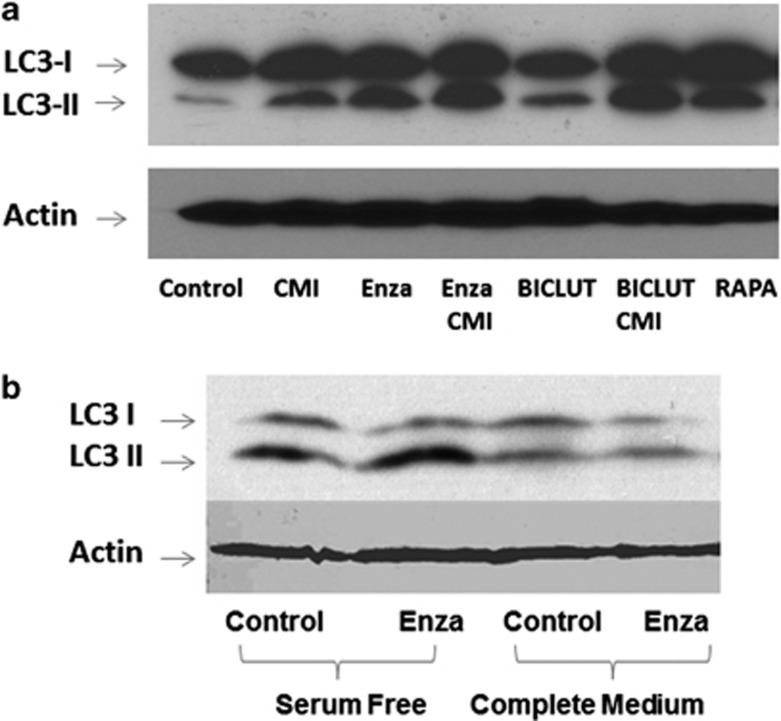
Antiandrogen-mediated autophagy is a class effect. (**a**) Representative western blotting analysis of LNCaP and C4-2B cell lines were treated with DMSO, 5 μM CMI, 10 μM ENZA, combination of CMI and ENZA, 10 μM bicalutamide and a combination of bicalutamide and CMI for 48 h, and 2 μM rapamycin for 4 h were used as a positive control. Cell lysates were harvested and subjected to western blotting analysis using autophagy markers, LC3-I and LC3-II. (**b**) Western blotting analysis of AR-negative PC3 cells treated with DMSO (vehicle control) and 10 μM ENZA in both normal serum and serum-free conditions.

To answer the question whether ENZA-mediated autophagy is a specific effect or can be seen with other ARSI therapy such as bicalutamide, we subjected LNCaP and C4-2B cells to both ENZA and bicalutamide treatment and examined for autophagic induction. Figure 2a shows representative western blotting analysis of LNCaP and C4-2B cell lines treated with DMSO (dimethyl sulfoxide; vehicle control), 5 μM CMI, 10 μM ENZA, combination of CMI and ENZA, 10 μM bicalutamide and a combination of bicalutamide and CMI for 48 h, and cells treated with 2 μM rapamycin for 4 h were used as a positive control. Both ARSI agents elicited autophagy as indicated by the transition of LC3-1 to LC3-II, comparable to that in the rapamycin-treated positive control. Addition of the late autophagy inhibitor CMI arrested the fusion of autophagosome to lysosome to the completion of autophagy and displayed even higher LC3-II/I ratios. To make certain that autophagy was induced via AR inhibition, PC3 cell line lacking detectable expression of AR were subjected to similar treatment under serum-free and full medium. As shown in Figure 2b, exposure of AR-negative PC3 cells to ENZA had no effect on the LC3-I to LC3-II conversion, suggesting that ENZA-mediated autophagy is specific to AR inhibition.

### AMPK is activated by AR inhibition

**Figure 3:**
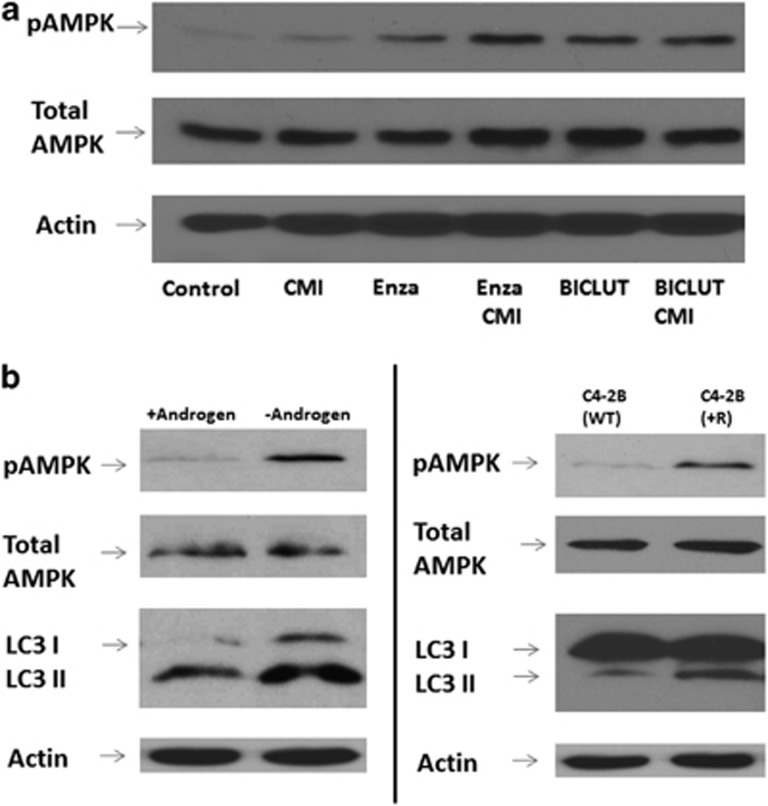
(**a**) AMPK is activated with AR blockage. LNCaP and C4-2B cell lines were treated with DMSO, 5 μM CMI, 10 μM ENZA, combination of CMI and ENZA, 10 μM bicalutamide and a combination of bicalutamide and autophagy inhibitor CMI for 48 h. Cell lysates were harvested and subjected to western blotting analysis using autophagy markers LC3-I and LC3-II and antibodies to phospho-AMPK and total AMPK. (**b**) Androgen deprivation and continuous androgen blockage by ENZA induces autophagy and activates AMPK phosphorylation. Left panel shows representative western analysis of C4-2B cells cultured under regular FBS (+Androgen) and charcoal-stripped FBS (−Androgen). Right panel shows similar analysis using ENZA-resistant cells (C4-2B+R) and their counterpart parental line (C4-2B-WT).

The activation of AMPK and the suppression of PI3K/AKT/mTOR signaling have been implicated in androgen deprivation-mediated autophagy.^7^ To determine the predominate mechanism involved in ARSI-mediated autophagy, we subjected C4-2B and LNCaP cells to both bicalutamide and ENZA treatment and analyzed for phosphorylated AMPK and AKT. As shown in Figure 3a, activation of AMPK significantly increased in cells treated with ARSI, whereas the level of phosphorylated AKT is minimally affected (data not shown). We next evaluated AMPK phosphorylation in cells that conferred resistant to ARSI, namely C4-2B and C4-2B+R cells. Under androgen deprivation (Figure 3b, left panel) or prolonged AR blockage with ENZA (right panel), the induction of autophagy was coupled with the activation AMPK, again suggesting that AMPK has a crucial role in the induction of autophagy.

### Knockdown of AMPK in LNCAP and C4-2B cells blocks the induction of autophagy

**Figure 4:**
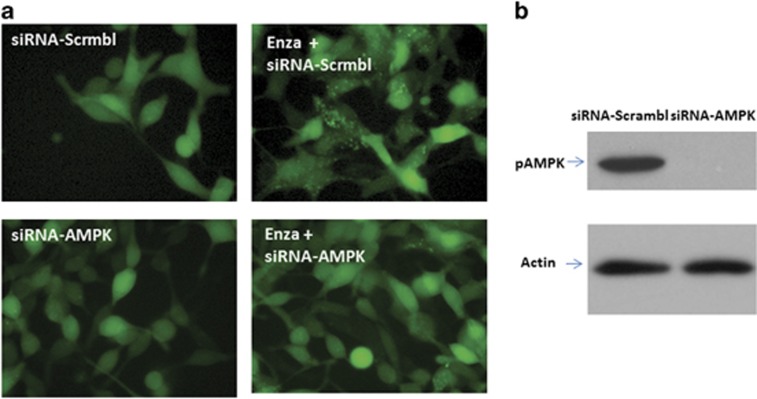
(**a**) Knockdown of AMPK in LNCAP-eGFP-LC3 cells blocks the induction of autophagy. LNCaP-eGFP-LC3 cells were transfected with negative control siRNA or siRNA targeting AMPK and treated with DMSO and 10 μM ENZA for 48 h and were then visualized by fluorescence microscopy. Shown are representative fluorescence microscopy of LNCaP-eGFP-LC3 with GFP-LC3 localization and puncta autophagosome formation represented by arrow. (**b**) Knockdown of AMPK was confirmed by western blotting. Immunoblot for cell lysates from LNCaP-eGFP-LC3 cells transfected with siRNA, indicating the knockdown of AMPK.

To prove the principle that activation of the AMPK pathway is responsible to the induction of autophagy mediated by ARSI, we used interference RNA to knock down the expression of AMPK in C4-2B cells and subsequently treated them with ENZA. Autophagy was not observed in cells with diminished level of AMPK expression as evidenced by the lack of green fluorescence punctate, compared with the bright punctate fluorescence in cells transfected with the scrambled control (Figure 4a). The same cells were subjected to western blotting analysis using anti-phospho-AMPK antibodies, verifying that AMPK is effectively knocked down (Figure 4b).

### AR signaling inhibition-induced autophagy is mediated through activation of AMPK activation and inhibition of mTOR signaling via Raptor

**Figure 5:**
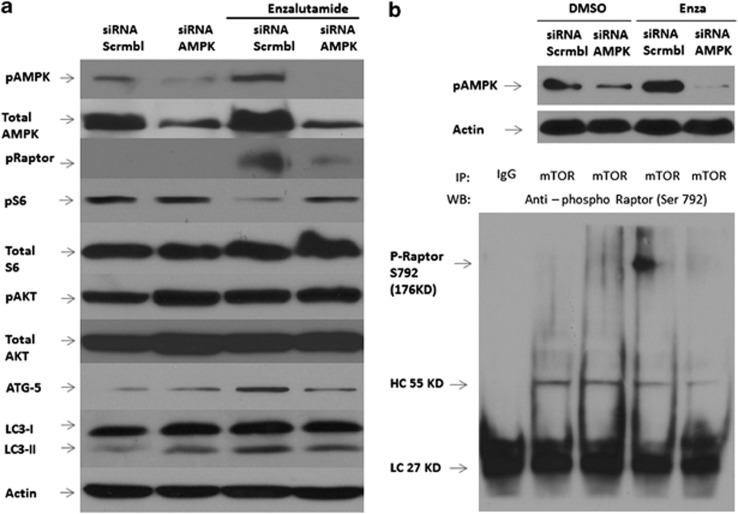
(**a**) Antiandrogen-induced autophagy is mediated through activation of AMPK activation and inhibition of mTOR signaling via Raptor. C4-2B cells were transfected with negative control siRNA or siRNA targeting AMPK and treated with DMSO and 10 μM ENZA for 72 h, and cell lysates were analyzed by immunoblotting with antibodies as indicated. Controls were treated with vehicle alone. β-Actin was detected as the loading control. (**b**) Under similar condition, C4-2B cells were incubated with (+) or without (−) ENZA for 72 h after transfection with siRNA, lysed and collected for immunoprecipitation (IP) with anti-mTOR antibody or with rabbit IgG as a control. Equal amounts of IP complex were loaded and resolved by SDS–PAGE and probed for the presence of phospho-Raptor at Serine 792 in the mTOR/Raptor immunocomplex with anti-phospho-Raptor polyclonal antibody, as shown in the bottom panel. The positions of heavy-chain (HC) and light-chain (LC) IgG were also indicated. The knockdown of AMPK in the cell lysate was further verified by blotting the total lysate with anti-phospho-AMPK antibody, as shown in the upper panel.

Previous studies demonstrated that the AMPK pathway directly interacts with TSC2/Raptor/mTOR complex to inhibit mTOR/S6K/4EBP signaling and the subsequent activation of autophagy.^11, 12, 13^ Cells with knockdown expression of AMPK were treated with vehicle and ENZA and then probed for phosphorylated Raptor, specifically detecting the phosphorylation of S792. As shown in Figure 5a, in the presence of ENZA and intact AMPK expression, phosphorylated Raptor level increased significantly, resulting in the consequential downregulation of pS6 and increased LC3-I to LC3-II conversion, whereas p-AKT remained unaffected. When AMPK was effectively knocked down, ENZA treatment did not affect the phospho-Raptor or phospho-S6 levels. These observations also correlated with reduced ATG-5 expression and reduced conversion of LC3-I to LC3-II. Hence, our data suggest the interaction between AMPK activation and suppression of mTOR via phosphorylation of Raptor at Serine 792 upon induction of ARSI-mediated autophagy. To confirm this interaction, we performed co-immunoprecipitation assays by pulling down the mTOR complex with anti-mTOR antibodies in the presence/absence of AMPK and ENZA. As shown in Figure 5b, we could only detect phospho-Raptor in the mTOR complex when AMPK was activated by ENZA treatment. Phospho-Raptor was undetectable in control conditions and when AMPK expression was knocked down, indicating a direct interaction between AMPK activation and mTOR suppression via phospho-Raptor in ARSI-induced autophagy.

### Knockdown of AMPK overcomes ENZA resistance

**Figure 6:**
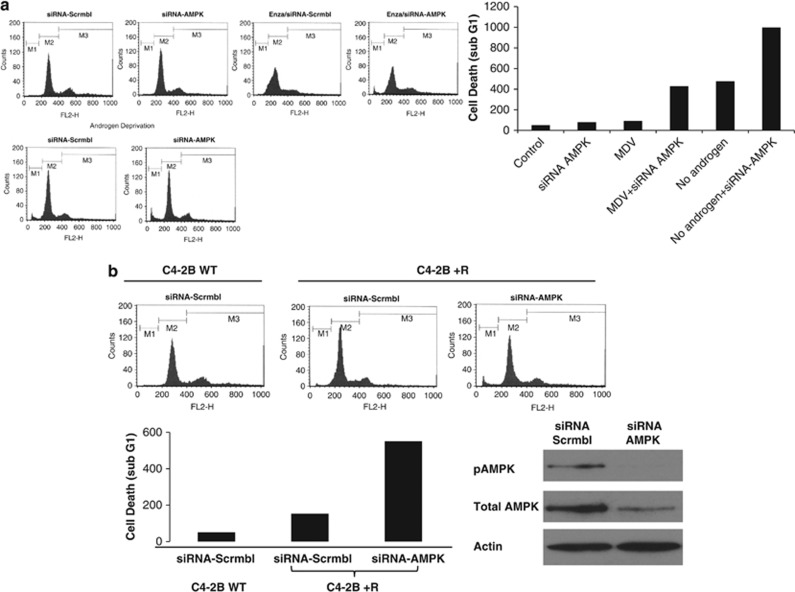
(**a**) Knockdown of AMPK sensitized C4-2B cells to ENZA-induced cell death. C4-2B cell death was assessed by propidium iodide (PI) staining and flow cytometry analysis after treatment with DMSO, 10 μM ENZA, AMPK siRNA or 10 μM ENZA plus AMPK siRNA for 72 h with and without androgen deprivation. sub-G1 content shown by fluorescence-activated cell sorting analysis representing cell death was plotted in the graph. (**b**) Knockdown of AMPK-sensitized cell death in ENZA-resistant C4-2B+R cells. Flow cytometry analysis using ENZA-resistant cells (C4-2B+R) and the parental line (C4-2B-WT), were subjected to transfection with AMPK siRNA or mock siRNA as negative control. Bottom panel showed quantification of sub-G1 population and confirmation of AMPK knock down by western blotting analysis of cell lysate 72 h after transfection.

As AMPK signaling appeared to be the upstream to ARSI-induced autophagy, we postulate that knocking down AMPK will effectively deprive the CRPC cells of their ability to undergo autophagy and promote apoptosis. LNCaP and C4-2B cells were transiently transfected with siRNA targeting AMPK and treated with ENZA. As shown in Figure 6a, cell cycle analysis using fluorescence-activated cell sorter indicated an increase in cell death, represented by the increased cell population in Sub-G1 phase. Similar findings were observed in the androgen-deprived condition. Next, we tested this hypothesis on ENZA-resistant cells C4-2B+R. Data in Figure 6b showed enhanced cell killing when AMPK was knocked down in the ENZA-resistant cells. The data support the notion that once the upstream signal for autophagy induction is suppressed, the ENZA-resistant cells become re-sensitized to ARSI-induced cell death.

### Inhibiting autophagy pharmacologically overcomes ENZA resistance and enhanced therapeutic response *in vitro* and *in vivo* using prostate xenograft mouse model

**Figure 7:**
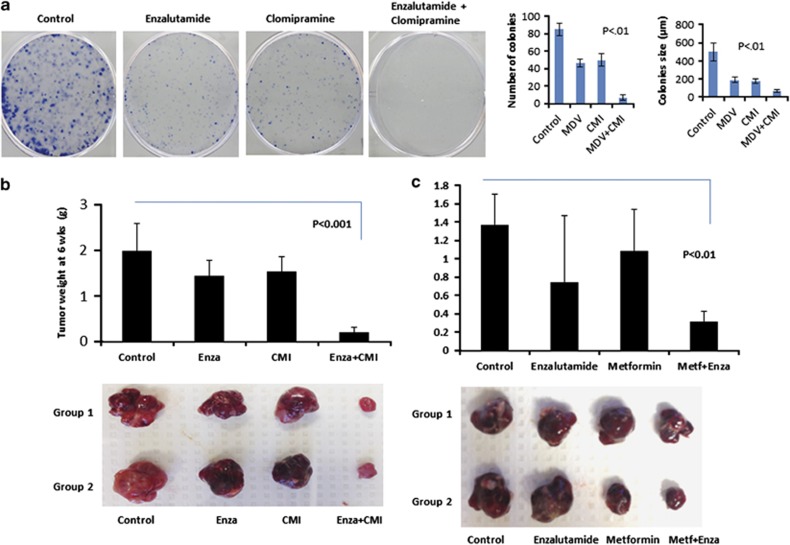
Inhibiting autophagy pharmacologically enhanced therapeutic response in ENZA-resistant cells *in vitro* and *in vivo* using prostate xenograft mouse model. (**a**) Blockage of antiandrogen-mediated autophagy decreases cells' ability to form colonies. C4-2B cells treated with DMSO, 5 μM CMI, 10 μM ENZA and a combination of CMI and ENZA were plated for the clonogenic assay to evaluate cell survival. Left panels showed quantification of the number of colonies and size of the colonies. Significant difference between the treatment groups were found using *t*-test (*P*<0.05). Values represent mean±s.e. (**b**) Inhibiting autophagy enhanced therapeutic response in ENZA-resistant tumors in prostate xenograft mouse model. ENZA-resistant cells (C4-2B+R, 1.5 × 10^6^ cells) were orthotopically implanted in four groups of SCID mice and randomly divided, with four mice in each group. Mice were treated after detectable PSA with: Control (vehicle only), ENZA, CMI, and ENZA plus CMI. At 6 weeks after implantation, mice were euthanized, and tumors were surgically dissected and weighted. The graph in the upper panel showed the average weight of tumors in each group. Lower panel showed two representative images of the tumors. (**c**) ENZA-resistant cells (C4-2B+R, 1.5 × 10^6^ cells) were orthotopically implanted in four groups of SCID mice and randomly divided, with 6–8 mice in each group. Mice were treated after PSA became detectable with: Control (vehicle only), ENZA (25 mg/kg), Metformin (300 mg/kg), and ENZA plus Metformin. At 4 weeks after implantation, mice were euthanized, and tumors were surgically dissected and weighed. The graph in the upper panel showed the average weight of tumors in each group. Lower panel showed two representative images of the tumors.

To provide an implication for therapeutic potential, we asked the question whether blocking autophagy would overcome ENZA resistance *in vitro* and *in vivo*. Clonogenic assays were used to evaluate cell ability to form colonies in the presence of an autophagy inhibitor CMI. CMI is an Food and Drug Administration (FDA)-approved drug to treat depression and has been shown to be a potent inhibitor of autophagy with little toxic affects both *in vitro* and *in vivo.*^14,15^ Colony formation in cells treated with ENZA or CMI alone was slightly reduced compared with control but was markedly impaired in the combined treatment. Their proliferative potential was also markedly reduced, based on the size of the colonies as shown in Figure 7a. To address our hypothesis that targeting autophagy could overcome resistant to ENZA therapy in CRPC in an *in vivo* model, we used SCID mice and orthotopically implanted ENZA-resistant cells into the prostate. Prostate-specific antigen (PSA) level was monitored until detectable around day 10, indicating tumor implantation. Treatments with control vehicles, CMI, ENZA and combination were dosed daily. At the end of 6 weeks after surgery, tumors were harvested and weighed. Mice treated with ENZA or CMI alone showed a 28% and 23% decrease in tumor size when compared with control mice, respectively. There were a significant reduction in tumor size by 91% in mice treated with ENZA in combination with the autophagy inhibitor CMI when compared with control mice, as shown in Figure 7b (*P*<0.001). Because of the elevated interest of metformin also as an autophagy modulator and its low toxicity and applicability, we conducted another *in vivo* study replacing CMI with metformin. Mice treated with ENZA or metformin produced marginally reduced tumor sizes than the control mice, while those treated with the combination of ENZA and metformin gave a drastic 78% reduction with a significant difference (*P*⩽0.01 by the Student's *t*-test).

## Discussion

The suppression of AR signaling continues to be important in optimizing therapy to treat or delay CRPC progression. However, CRPC cells are able to adapt and exploit survival mechanisms to counter the effects of ARSI, thus rendering the therapy ineffective after the initial response.^10,16, 17, 18^ In this report, we describe the novel observation that CRPC cells resort to autophagy as an escape mechanism to evade ARSI, even with the new generation therapy, ENZA. Recent studies suggested that autophagy has a pro-survival role in cells subjected to ARSI through AR knock down, androgen deprivation or by bicalutamide in androgen-responsive cell line LNCaP.^6,9,19^ Our result is consistent with these studies but also furthers the finding in other AR-positive CRPC cells, including CWR22Rv1 and C4-2B. Furthermore, we report CRPC cells that conferred ENZA resistance display a higher level of autophagy than control cells, implicating that autophagy is not only a survival mechanism but also is associated with chemoresistance. The autophagic cancer cells' ability to evade apoptosis is vital to drug resistance, but the mechanism remains speculative and warrants further investigation.

**Figure 8:**
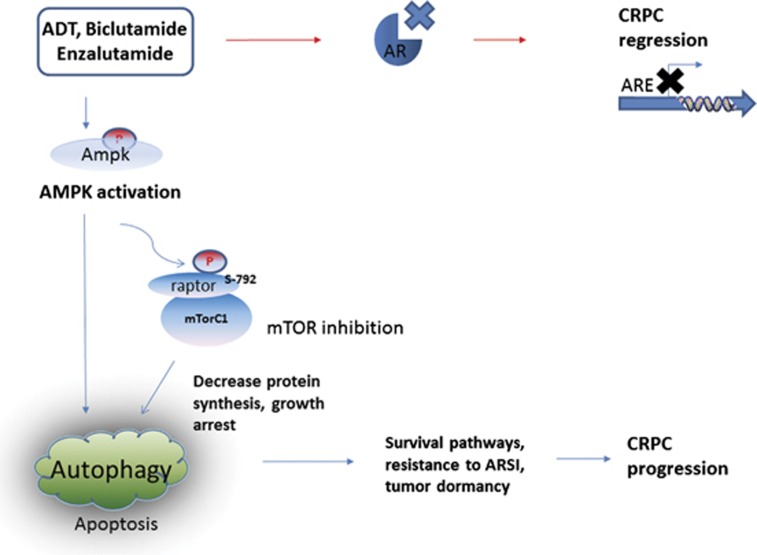
Potential mechanism in autophagy activation as a survival mechanism to evade cancer therapies by ARSI in CRPC.

Regulation of autophagy is complex and often involves multiple pathways, including the suppression of PI3K/AKT/mTOR and the activation of AMPK pathways through direct stimulation of ULK1.^20,21^ Interaction between AMPK and mTORC1 upon induction of autophagy has been described previously in other cell types.^11,12^ Specifically, active AMPK directly phosphorylates the TSC2 tumor suppressor, resulting in the inactivation of Rheb and sequential inhibition of the mTORC1 kinase.^13^ Alternatively, activated AMPK can also directly phosphorylate Raptor (one of the four subunits of the mTORC1 complex) at two conserved sites Ser722 and Ser792 to suppress the mTORC1 complex.^12^ Raptor is responsible for the recruitment of 4EBP1 and the p70 ribosomal S6 kinase (p70S6K1) to the mTORC1 complex.^20,21^ Boutin *et al.*^9^ reported that pAKT is slightly downregulated by 21±18%, whereas the phosphorylation of p70S6K downstream signal is much more at 44±23%.^10^ Previous reports suggested that when AR axis is suppressed in the setting of *Pten* loss, prostate cells resorted to reciprocal negative feedback to activate PI3K or mitogen-activated protein kinase pathway to adapt and survive and allow cancer to progress.^22,23^ However, in our experiments, we did not see a significant change of AKT signaling pathway, but we saw a much more pronounced downregulation of the mTOR downstream target, pS6. Neither was there a significant change in mTOR phosphorylation upon autophagy induction by ARSI (data not shown). We hypothesize that the AKT pathway is not the predominant regulator of ARSI-induced autophagy, whereas AMPK activation has a significant role. Interfering with AR signaling axis here only elicits autophagy without PI3K crosstalk as phosphorylation of both AKT and mTOR was unaltered. In the current study, we demonstrated that AMPK was clearly activated in ARSI-induced autophagy. siRNA targeting AMPK prevented autophagy induction when exposed to ARSI. We further showed evidence that AMPK activation led to phosphorylation of Raptor at Serine 792 in the mTOR complex resulting in the sequential suppression of its downstream signaling, indicated by the reduced activation of pS6 in cells with ARSI-mediated autophagy. The mechanistic evidence in this report suggested that targeting AMPK pathway could be an important adjunct to prostate cancer therapy as illustrated in Figure 8. Androgen-deprivation therapy using ARSI including bicalutamide and ENZA may halt CaP progression even in CRPC patients. Nonetheless, activation of AMPK mediated by ARSI may provide the tumor cells a survival mechanism via autophagy that leads to CRPC progression.

In this report, we show novel evidence that both naive and ENZA-resistant CRPC cell lines are more prone to cell death, exhibit impaired clonogenic ability and re-sensitized to ENZA therapy when exposed to combination treatment with autophagy inhibitor CMI. Autophagy inhibitors, particularly chloroquine and 3-methyladenine, have been shown to sensitize cancer cells to tamoxifen, cisplatin, ionizing radiation or anti-angiogenic treatment^5,24, 25, 26, 27, 28^ and have been used in numerous ongoing clinical trials as adjunctive therapy or primary therapy for breast, lung, prostate, pancreatic and skin cancer.^3^ Currently, the only FDA-approved agents that are able to inhibit autophagy are chloroquine, an antimalarial drug, and its derivative hydroxychloroquine. One completed clinical trial using hydroxychloroquine suggested a survival advantage when added to conventional treatment for glioblastoma multiforme, but the result was not statistically significant.^29^ The lack of significance could be due to small sample size or that hydroxychloroquine failed to sufficiently inhibit autophagy in patients. There is a need for pre-clinical investigation to develop and evaluate new autophagy inhibitors as a therapeutic strategy in cancer beyond chloroquine. Here, we reported that CMI, an FDA-approved antidepressant, is effective in inhibiting autophagy and enhanced therapeutic response in ENZA-resistant cells *in vitro* and *in vivo* using the orthotopic xenograft model combined with ENZA. Complimentary to this finding, we also tested metformin, the FDA-approved anti-diabetic drug, using the same animal model. Both drugs increased the efficacy of ENZA for the CRPC cell line. Previous studies have showed that metformin is a potent autophagy modulator through AMPK and mTOR signaling.^30, 31, 32^ In addition, metformin inhibited 2-deoxyglucose-induced autophagy in prostate cancer cells.^33^ Treatment with metformin alone or in combination with mTOR inhibitors have been shown to be effective in melanoma, lymphoma and breast cancer.^31,32,34^ This combined treatment, an equivalent to acute nutrient depletion may activate the eukaryotic elongation factor 2 kinase (eEF2K) and block mRNA translation elongation.^35^ Further exploring of the AMPK–eEF2K axis in the ARSI-treated cells may be intriguing. We provided an additional rationale for clinical trials using CMI and metformin as alternatives to chloroquine in metastatic prostate cancer. Furthermore, autophagic inhibition could also have a role in enhancing therapeutic effects on bone metastasis. Experiments are underway in our labs to study the effects of autophagy inhibition in delaying or attenuating bone metastasis in CRPC.

Most cancer therapies induce autophagy as a cytoprotective stress response and subsequently lead to the attenuation of their efficacy. The lack of a complete or lasting response of most monotherapies, including tyrosine kinase inhibitors, allosteric mTOR inhibitor (Everolimus) or the latest generation antiandrogen, may be explained by the pro-survival function of autophagy. The critical question is whether the dual roles of autophagy as pro-survival or pro-death can both be exploited to improve cancer therapy in advanced CRPC. At the same time, therapies that induced excessive autophagy to trigger pro-death properties may not be well tolerated by patients owing to increased dosage requirement. Here, we provide preclinical data to support that targeting autophagy could be an attractive option to exploit, especially with autophagy modulators that has been proven to be safe. Both CMI and metformin have been in clinical use since 1960, with established safety records and well tolerated by patients. To potentially give CRPC patients who progress while on ENZA therapy a more durable response, combining a well-tolerated and safe autophagy modulator with ENZA therapy maybe warranted in a clinical trial.

In summary, our data support autophagy as an adaptive response to ARSI and an important mechanism of resistance to the new generation ARSI, ENZA. ARSI-mediated autophagy is dependent on the activation of AMPK pathway and the suppression of mTOR downstream signaling via phosphorylation of Raptor. Blocking autophagy pharmacologically or genetically significantly impairs prostate cancer cell survival *in vitro* and *in vivo*, implying the therapeutic potential of autophagy inhibitors in the antiandrogen-resistance setting.

## Materials and methods

### Cell lines and reagents

LNCaP, CWR22Rv1 and PC-3 prostate cancer cell line was obtained from the American Type Culture Collection (Manassas, VA, USA). Cells were cultured in RPMI supplemented with 10% fetal bovine serum (FBS) and penicillin–streptomycin (100 IU/ml and 100 μg/ml, respectively) or 5% charcoal-dextran-stripped FBS (CS-FBS) and penicillin/streptomycin in 37 °C and 5% CO_2_ atmosphere. LNCaP passage numbers <20 were used throughout the study. All experiments with cell lines were performed within 6 months of receipt from ATCC or resuscitation after cryopreservation. C4-2B cells were kindly provided and authenticated by Dr Leland Chung, Cedars-Sinai Medical Center, Los Angeles, CA, USA. The generation of stably overexpressing eGFP-LC3 fusion gene in LNCaP, CWR22Rv1 and PC3 cells (LNCaP-eGFP-LC3, CWR22-eGFP-LC3 and PC3-eGFP-LC3, respectively) were described previously.^2^

### Cell cycle analysis

In each experiment, a sample of 10 000 cells was subjected to DNA staining with propidium iodide to evaluate cell cycle distribution, using flow cytometry analysis. Briefly, cells were trypsinized, washed and fixed in phosphate-buffered saline with 1% formaldehyde for 10 min and subsequently stained with propidium iodide for analysis by a FACScan system and CellQuest program (Becton Dickinson, San Jose, CA, USA). LNCaP and C4-2B cells transfected with AMPK siRNA (Ambion, Foster City, CA, USA) or negative control (scrmbl) were treated with ENZA the next day and harvested 72 h post transfection for FACScan analysis for sub-G1 content.

### Generation of ENZA-resistant CaP cells and RNA deep sequencing

ENZA-resistant LNCaP C4-2B cells were established via culture in media containing increasing dosage of ENZA up to 40 μM over a 4-month period. Cells were then maintained in medium containing 20 μM of ENZA. Total RNA was extracted from C4-2B ENZA-resistant cells (C4-2B+R) and parental cells (C4-2B-WT) and then submitted for deep RNA sequencing and bioinformatics analysis on Illumina HiSeq 2000 (DNA Technologies Core at the UC Davis Genome Center, using the Illumina sequencing platforms (HiSeq 2500), Sacramento, CA, USA): 100-bp, paired end sequencing multiplexed 4 samples/lane. The levels of genes expression are expressed in FPKM where 3 FPKM=1 transcript/cell.

### Cell extraction, immunoprecipitation and immunoblotting

Cell lysates were collected in radio-immunopreicipitation assay buffer and subjected to western blotting analysis (40 μg/lane) as described previously.^36,37^ For immunoprecipitation, 500 μg of soluble protein was first incubated with primary antibodies for 2 h at room temperature and further incubated overnight at 4 °C after addition of 25 μl of protein A/G-Sepharose beads (Santa Cruz Biotechnology, Santa Cruz, CA, USA). The immunoprecipitated proteins were properly washed and separated by SDS–PAGE (sodium dodecyl sulfate–polyacrylamide gel electrophoresis) western blotting analysis. The following antibodies were used in this study: β-actin (Sigma-Aldrich, St Louis, MO, USA), ATG-5 (Santa Cruz Biotechnology), phospho-Src (Tyr416), Src, LC3, phospho-Akt (Ser473), Akt, phospho-mTOR (Ser2448), mTOR, phospho-S6 (Ser235/236), S6, phospho-AMPKα (Thr172), phosphor-Raptor (Serine 792), and AMPKα (Cell Signaling Technology, Beverly, MA, USA).

### Fluorescent microscopy

LNCaP-eGFP-LC3, CWR22Rv1-eGFP-LC3 and PC3-eGFP-LC3 were seeded on coverslips and treated with 10 μM ENZA for 48 h or 2 μM rapamycin for 4 h as positive control, followed by 4% paraformaldehyde fixation and mounting with SlowFade with 4,6-diamidino-2-phenylindole (Invitrogen, Carlsbad, CA, USA). eGFP-LC3 and acridine orange staining for autophagosomes were examined under a × 40 lens (excitation, 488 nm; emission, 535 nm).

### Clonogenic assay

LNCaP and C4-2B cells and C4-2B+R (ENZA resistant) were treated with DMSO (vehicle control), 5 μM CMI, 10 μM ENZA and a combination of CMI and ENZA and used clonogenic assay to evaluate the cells' ability to form colonies as we described elsewhere.^36,37^ In brief, cells were treated with 1 × trypsin for 5 min in a 37 °C incubator and pipetted several times so that most cells were in single-cell forms. Five thousand cells were plated on to a 35-mm six-well plate. The covering medium was changed every 2 or 3 days during culture. After 20 days, cultures were fixed and stained with crystal violet solution (10% acetic acid, 10% ethanol and 0.06% crystal) and then visualized using an Olympus IX70 microscope (with × 4 and × 40 objectives). The number of colonies formed was counted for each well, approximately containing more than three cells to be counted. The average of counts from three random fields for each well was taken as the colony number.

### *In vivo* tumor biology

Animal studies were conducted in accordance with institutional ethical guidelines for the care and use of experimental animals. In all, 1.5 × 10^6^ C4-2B+R (ENZA resistant) cells co-suspended with 30% matrigel were injected orthotopically into male SCID mice. The PSA level was checked every 7 days, and treatment began when PSA first become detectable, indication tumor implantation. Sixteen mice were randomly divided into four groups and treated with control vehicle, CMI (10 mg/kg/day), metformin (300 mg/kg/day) and ENZA (25 mg/kg/day) or the combinations of Enza+CMI and Enza+metformin via esophageal gavaging. At the end of 6 weeks, mice were killed, and their prostates were collected for weight and pathological analyses. PSA levels were determined by PSA (human) ELISA kit (Abnova, Taipei, Taiwan).

### Statistical analysis

Data are shown as the mean±s.d. All were from at least three independent experiments and subjected to paired *t*-tests and three-way analysis of variance. *P*<0.05 was considered significant.

## Notes

There is potential conflict of interest.
